# Incidence and risk factors associated with pressure injury in patients with traumatic brain injury

**DOI:** 10.1111/ijn.12821

**Published:** 2020-01-29

**Authors:** Sibila Lilian Osis, Solange Diccini

**Affiliations:** ^1^ School of Nursing State University of Amazonas, Brazilian Association Critical Care Nurses Manaus Brazil; ^2^ School of Nursing Federal University of São Paulo São Paulo Brazil

**Keywords:** nursing, pressure injury, pressure ulcer, risk factors, traumatic brain injuries

## Abstract

**Aim:**

To identify the prevalence of pressure injury in patients diagnosed with traumatic brain injury and analyse the risk factors involved during hospitalization.

**Methods:**

This was a prospective study evaluating patients who were diagnosed with traumatic brain injury between November 2013 and September 2014. Patient characteristics, clinical and metabolic factors and therapeutic interventions, were evaluated within 30 days of hospital admission.

**Results:**

Most of the 240 patients included in the study were male, young, and non‐Caucasian. The incidence of pressure injury was 18.8%. In terms of severity classification, the incidence of pressure injury was 2.7%, 23.2%, and 42.6% in mild, moderate, and severe traumatic brain injury, respectively. Pressure injury development was more likely in the first 10 days of hospitalization. A moderate or severe traumatic brain injury classification, the use of noradrenaline, and older age were pressure injury risk factors. The presence of pressure injury was associated with mortality within 30 days of hospitalization (*P* < .001).

**Conclusion:**

The incidence of pressure injury was high in patients diagnosed with traumatic brain injury, especially in those whose injury was classified as severe. Older age, noradrenaline use, and a classification of moderate or severe traumatic brain injury were identified as pressure injury risk factors.

## SUMMARY STATEMENT

What is already known about this topic?

- Traumatic brain injury is a major cause of disability in young adults.
- One of the complications reported in patients diagnosed with traumatic brain injury is pressure injury. Complications related to pressure injury may increase the risk of mortality.
- Pressure injury occurs because of a variety of factors, such as changes to skin perfusion, metabolism, nutrition, body temperature, mobility, and sensory perception.

What this paper adds?

- A severity classification of moderate to severe traumatic brain injury increases the risk of developing pressure injuries.
- Older age and the use of noradrenaline, as well as the severity of the traumatic brain injury classification, were identified as risk factors for the development of pressure injury.

The implications of this paper:

- To prevent the onset of pressure injury in patients with head trauma, nurses should be aware of the risk factors that increase the likelihood of developing these lesions.
- Once the patient has been admitted to the emergency room with cranial trauma, the risk of developing pressure injury must be evaluated, especially in patients with moderate to severe head trauma.
- Patients classified as having moderate or severe head injury, aged over 60 years, and in need of noradrenaline should receive specific attention and intervention related to preventing pressure injuries.

## 1 INTRODUCTION

Traumatic brain injury (TBI) is defined as an injury to the brain caused by external physical force. TBI is considered to be a silent epidemic (Center for Disease Control and Prevention, 2010) and is a major cause of disability and death in young adults (Andriessen et al., 2011; Center for Disease Control and Prevention, 2010; Coronado et al., 2011; Majdan et al., 2013). Its economic consequences include the direct financial costs of hospitalization and rehabilitation, as well as indirect costs such as loss of life years and low posttraumatic productivity. Social costs are considered incalculable and are related not only to death but also to a reduction in quality of life, affecting both the patient and their loved ones (World Health Organization, 2006).

TBI severity can be classified by the application of scales and/or neuroimaging tests. TBI severity classification assists in accurate diagnosis, grouping patients with the same characteristics, and predicting outcomes (Subhas & Appleby, 2011; World Health Organization, 2006). The Glasgow Coma Scale (GCS) classifies TBI as mild, moderate, or severe (Teasdale & Jennett, 1974). Morgado and Rossi (2011) have identified that most patients are classified as having mild TBI (82.4%), followed by severe (15.6%), and then finally moderate (2.0%) (Morgado & Rossi, 2011). Although the percentage of patients classified with moderate or severe TBI is small, this population develops more complications and has higher risk of mortality during the hospitalization phase (Andriessen et al., 2011).

One complication in patients with diagnostic TBI is pressure injury (PI), also referred to as pressure ulcer, which has a reported incidence of 16% (Dhandapani, Dhandapani, Agarwal, & Mahapatra, 2014) to 26% (Zampolini, Zaccaria, Tolli, Frustaci,, & Franceschini, 2012). PIs are considered a challenge to the health professional (Dealey et al., 2015) and occur because of a variety of factors, such as changes in skin perfusion (Cox, 2013), metabolism (Coleman et al., 2013), nutrition (Cox & Rasmussen, 2014; Dhandapani et al., 2014), body temperature (Coleman et al., 2013; Cox & Rasmussen, 2014), and mobility and sensory perception (Bergstrom, Demuth, & Braden, 1987). The use of vasopressors (Cox, 2013), mechanical ventilation (Manzano et al., 2010), prolonged hospitalization time (Szubski et al., 2014), and surgical procedures (Scarlatti, Michel, Gamba, & de Gutierrez, 2011) are also factors that may be associated with PI development. Patients diagnosed with TBI exhibit several factors that can contribute to PI during hospitalization.

In severe TBI, PI has been associated with decreased haemoglobin and the initiation of enteral feeding after the seventh day of hospitalization. Mortality in patients with PI has been recorded at 64%, which is five times higher than that in patients without PI (Dhandapani et al., 2014). However, risk factors that contribute to PI development in hospitalized patients with TBI diagnoses are not yet fully understood.

## 2 METHODS

### 2.1 Aim

The aim of this study was to identify the prevalence of PI in patients with a TBI diagnosis and to analyse the risk factors involved during hospitalization. Data collection was performed between November 2013 and September 2014.

### 2.2 Design

This was a prospective study that was carried out in a neurotrauma reference hospital in the city of Manaus, in the state of Amazonas (AM), Brazil.

### 2.3 Sample/Participants

The sample was calculated from a population of 360 patients diagnosed with TBI admitted between January and June 2013 at the same institution. The estimated proportion of patients with PI was 20%, based on Brazilian studies with rates ranging from 10% (Rogenski & Santos, 2005) to 39% (Crozeta, 2009). A confidence interval of 95% and a sampling error of 3% were adopted. Therefore, the calculated sample size was 240 patients.

**Figure 1:**
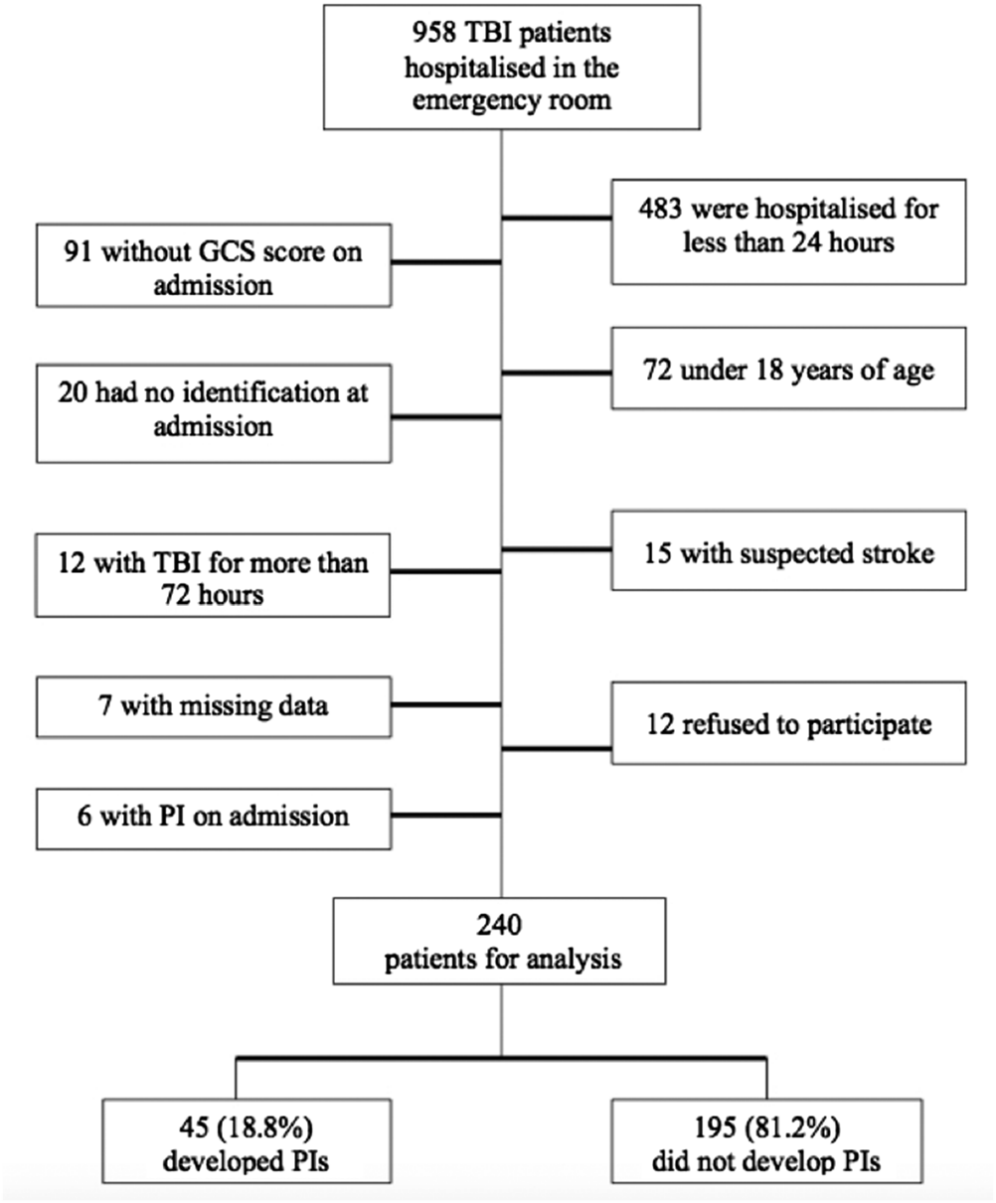
Flow diagram of TBI patients selected for the study

Inclusion criteria were patients with diagnostic TBI in the emergency room, aged over 18 years, with emergency room records of GCS, and with lengths of stay of greater than 24 hours. Patients in the emergency room with PI or traumatic skin injuries in PI risk areas or with stroke, spinal cord injury, or a trauma history of more than 72 hours were excluded. A total of 958 patients with TBI diagnoses were evaluated during the study period. Figure 1 describes the included and excluded patients.

### 2.4 Variables

The variables collected at hospital admission were sex, age, skin colour, comorbidities, number of intracranial injuries identified by CT scan, GCS value, TBI severity classification based on hospital admission via the GCS score (Mild, 15‐13 points; Moderate, 12‐9 points; Severe, 8 or fewer points) (Teasdale & Jennett, 1974), Braden Scale value, the total Braden Scale risk classification score (Without Risk, 19‐23 points; At Risk, 15‐18 points; Moderate Risk, 13‐14 points; High Risk, 10‐12 points; Very High Risk, 9 or fewer points) (Bergstrom et al., 1987), treatment type (medical or surgical), body mass index (BMI), temperature (°C), systolic blood pressure (SBP), diastolic blood pressure, mean arterial pressure, and haematocrit, haemoglobin, urea, creatinine, albumin, and glucose levels (Coleman et al., 2013; Cox, 2011; Fife et al., 2001; Flattau & Blank, 2014; Posthauer, Banks, Dorner, & Schols, 2015). Variables such as location of PI, number of PI, stage of PI, mechanical ventilation, sedation, noradrenaline use, intracranial pressure (ICP) catheters, absolute rest time, length of intensive care unit (ICU) stay, and outcome (discharge or death) were evaluated during hospitalization. In the National Pressure Ulcer Advisory Panel, classification was used to define the PI stage (National Pressure Ulcer Advisory Panel, 2007).

### 2.5 Data collection

Data were collected by the researcher within the first 30 days of patient hospitalization. An active search for hospitalized patients diagnosed with TBI was performed daily in the surgical emergency room. The researcher's phone was available to the emergency team. Individual assessments were conducted by the researcher daily by evaluating the patients included in the research.

Information regarding patient history and comorbidities was acquired from the patient or family member. Information regarding hospital admission, admission GCS value, vital signs, invasive monitoring, medications used, mechanical ventilation, imaging results, and surgical procedures were extracted from the medical record. At each visit, the researcher applied the GCS and Braden Scale and performed the Braden Scale risk classification. The researcher performed the collection of blood tests, anthropometric measures for weight estimation, and BMI calculation at admission and every 7 days. A daily skin inspection was performed to identify PI and staging. The implementation and application of PI prevention protocol were performed by the unit nursing staff according to hospital protocols.

A pilot study for the validation of the data collection instrument was performed with 10 patients from the same research institution, as it is the only neurotrauma referral hospital located in the State of Amazonas. After adjusting the analysed variables, the data collection began.

### 2.6 Ethical considerations

The project was approved by the Research Ethics Committee of the Federal University of São Paulo (No. 422.917). All patients or their families gave their informed consent prior to their inclusion in the study.

### 2.7 Data analysis

The Statistical Package for Social Sciences program, version 22.0, was used to analyse the results. Data were characterised using absolute and relative frequencies and central tendency and dispersion measures. PI incidence was presented as a relative frequency. Pearson chi‐square test was used to evaluate the different categories. The nonparametric Mann‐Whitney test was used to compare medians between groups. A times‐to‐event curve was plotted for the occurrence of PI using the Kaplan‐Meier method. A value of *P* < .05 was considered statistically significant. A multivariate model was developed to study the independent variables associated with PI development. Initially, bivariate analyses were plotted. A multivariate binary logistic model was created using a stepwise method, and independent variables with *P* < .2 were used for the final model. The Hosmer‐Lemeshow test was applied to evaluate the model fit, which was considered good if *P* ≥ .05.

## 3 RESULTS

### 3.1 Participants and prevalence of pressure injuries

**Figure 2:**
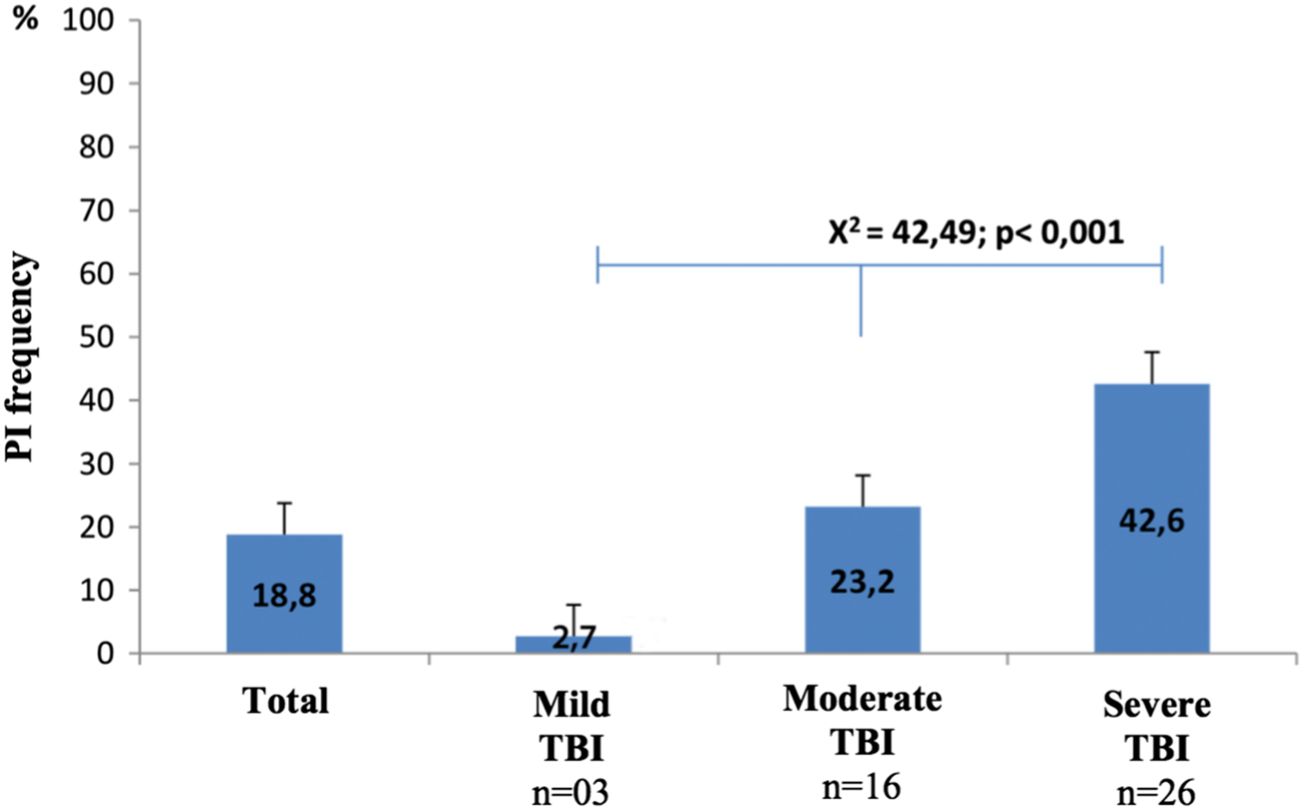
Frequency of PI in patients diagnosed with TBI classified by severity

The sample size was 240 patients. They were classified as mild TBI 110 (45.8%), moderate 69 (28.8%), and 61 (25.4%) severe. Table 1 summarizes the demographic and clinical characteristics of patients with and without PIs at hospital admission. Patients in the two analysed groups were predominantly male, young, and non‐white. Forty‐five (18.8%) of the evaluated patients developed 53 PIs, with the largest frequency in the groups classified as moderate or severe TBI (Figure 2).

**Table 1:** Demographic and clinical characteristics of patients

	Patients				
Variable	With PI n (%)	Without PI n (%)	Test	Value Test	*P*
Age, y					
Median (min‐max)	33 (18‐85)	30 (18‐79)	*U* Mann‐Whitney (*Z* test)	3.63 (−1.80)	.07
Sex					
Male	42 (20.2)	166 (79.8)	Likelihood ratio test	2.45	.12
Female	3 (9.4)	29 (90.6)			
Skin colour					
White	9 (29.0)	22 (71.0)	Likelihood ratio test	2.24	.13
Non‐white	36 (17.2)	173 (82.8)			
Comorbidities					
Yes	5 (27.8)	13 (72.2)	Likelihood ratio test	0.94	.33
No	40 (18.0)	182 (82.0)			
BMI					
Normal	29 (20.4)	113 (79.6)		—	1.0
Underweight	5 (16.1)	26 (86.7)	Likelihood ratio test	0.30	.58
Overweight	10 (18.2)	45 (81.8)	Likelihood ratio test	0.12	.72
Obese	1 (08.3)	11 (91.7)	Likelihood ratio test	1.23	.27
GCS					
Median (min‐max)	8 (3‐15)	13 (3‐15)	*U* Mann‐Whitney (*Z* test)	1.83 (−6.13)	.07
Braden Scale					
Median (min‐max)	9 (6‐16)	14 (8‐23)	*U* Mann‐Whitney (*Z* test)	270 (−3.17)	<.001*
Mild TBI	11 (9‐15)	14 (9‐23)	*U* Mann‐Whitney (*Z* test)	78 (−1.54)	.14
Moderate TBI	9 (9‐16)	13 (9‐18)	*U* Mann‐Whitney (*Z* test)	213 (−3.04)	.002*
Severe TBI	9 (6‐13)	9 (8‐17)	*U* Mann‐Whitney (*Z* test)	270 (−3.17)	.001*
Risk‐Braden Scale					
Mild	2 (3.4)	57 (96.6)	—	—	1.0
Moderate	5 (6.0)	78 (94.0)	Likelihood ratio test	0.53	.46
High	4 (11.8)	30 (88.2)	Likelihood ratio test	2.39	.12
Elevated	34 (53.1)	30 (46.9)	Likelihood ratio test	42.77	<.001*
Clinical (median, min‐max)					
Temperature, °C	35.8 (33.8‐37.6)	36.0 (34.3‐37.5)	*U* Mann‐Whitney (*Z* test)	3.47 (−2.22)	.25
SBP, mmHg	130 (85‐190)	130 (80‐230)	*U* Mann‐Whitney (*Z* test)	4.13 (−0.59)	.55
DBP, mmHg	80 (39‐120)	80 (30‐140)	*U* Mann‐Whitney (*Z* test)	3.86 (−1.24)	.21
MAP, mmHg	96.6 (54.3‐143.3)	93.3 (50‐170)	*U* Mann‐Whitney (*Z* test)	4.25 (−0.30)	.75
Albumin, g/dL	3.6 (1‐4.6)	3.9 (2‐5.3)	*U* Mann‐Whitney (*Z* test)	3.02 (−3.25)	<.01*
Haematocrit, %	36.6 (23.8‐48.2)	39.7 (13‐50)	*U* Mann‐Whitney (*Z* test)	3.33 (−2.50)	.01*
Glycaemia, mg/dL	117.5 (21‐526)	113 (21.9‐526)	*U* Mann‐Whitney (*Z* test)	2.90 (−3.52)	<.01*
Urea, mg/dL	24 (9.9‐154)	22 (5‐75)	*U* Mann‐Whitney (*Z* test)	3.708(−1.62)	.10
Creatinine, mg/dL	0.7 (0.4‐23)	0.7 (0.3‐3.2)	*U* Mann‐Whitney (*Z* test)	3.66 (−1.75)	.08

Abbreviations: BMI, body mass index; DBP, diastolic blood pressure; MAP, mean blood pressure; PI, pressure injury; SBP, systolic blood pressure; TBI, traumatic brain injury.

* Statistically significant.

### 3.2 Risk factors

The most‐identified comorbidity was hypertension, which was found in 11 (4.6%) patients, of whom four (8.9%) were in the PI group and seven (3.6%) in the non‐PI group. Six (2.5%) patients had other comorbidities, and one (20%) PI group patient had diabetes mellitus.

An evaluation of intracranial injuries in the PI group revealed that 23 (51.1%) patients had one intracranial injury, 16 (22.9%) had two injuries, and six (24%) had three or more injuries. Among patients without PI, 122 (62.6%) had one injury, 54 (77.1%) had two injuries, and 19 (76%) patients had three or more intracranial injuries. There was no statistical significance for the difference between the groups (*U* Mann‐Whitney [*Z* test] = 3.85 [−1.45]; *P* = .15).

The following variables occurred statistically significantly more often in the PI group: severe or moderate TBI classification, low Braden Scale score/high Braden Scale risk classification, changes in haematocrit albumin and glucose values, and surgical treatment. Median Braden Scale was lower in patients with moderate to severe TBI who developed PI, a relationship that was significant compared with that for patients without PI. None of the seven (2.9%) patients who were classified by the Braden Scale as being not at risk developed PI. Regarding treatment type, patients who underwent surgery were more likely to suffer PI.

The factors related to treatment, mechanical ventilation, sedation, noradrenaline, minimum stimulation time, and the risk of developing PI are presented in Table 2.

**Table 2:** Risk factors related to PI development

	Patients				Test		
Factor	With PI n (%)	Without PI n (%)	Odds Ratio	Confidence Interval 95%		Value Test	*P*
Treatment							
Surgery	23 (29.1)	56 (70.9)	2.59	(1.34‐5.03)	Likelihood ratio test	7.91	.004
Clinic	22 (13.7)	139 (86.3)					
Mechanical ventilation							
Yes	42 (43.3)	55 (56.7)	35.6	(10.60‐119.76)	Likelihood ratio test	69.79	<.001
No	03 (2.1)	140 (97.9)					
Sedation							
Yes	39 (44.3)	49 (55.7)	19.3	(7.73‐48.52)	Likelihood ratio test	60.23	<.001
No	06 (3.9)	146 (96.1)					
Norepinephrine							
Yes	32 (45.1)	39 (54.9)	9.8	(4.72‐20.51)	Likelihood ratio test	42.24	<.001
No	13 (7.7)	156 (92.3)					
Time minimal stimulation							
Days	3 (1‐13)	2 (0‐11)	—	—	*U* Mann‐Whitney (*Z* test)	2.89 (‐3.73)	<.001
Median (min‐max)							

Of the 45 patients with PI, 23 (28.8%) developed the injury in the ICU, 10 (22.2%) patients developed PI during hospitalization in the emergency surgery unit, six (13.3%) developed PI in the surgical centre, and six (13.3%) developed PI in the admissions unit. Of the 80 patients who were hospitalized in the ICU, 12 (15.0%) were admitted with PI, of whom six (50.0%) were hospitalized in the surgical centre and six (50.0%) in the emergency surgery unit. The median length of ICU stay was 11 (2‐42) days in the PI group and 9 (1‐29) days in the non‐PI group (*U* Mann‐Whitney [*Z* test] = 2.22 [−6.14]; *P ≤* .001). An ICP monitoring catheter was used in seven (2.9%) patients, two (28.6%) in the PI group, and five (71.4%) in the non‐PI group (Likelihood ratio test = 0.74; *P* = .40).

**Figure 3:**
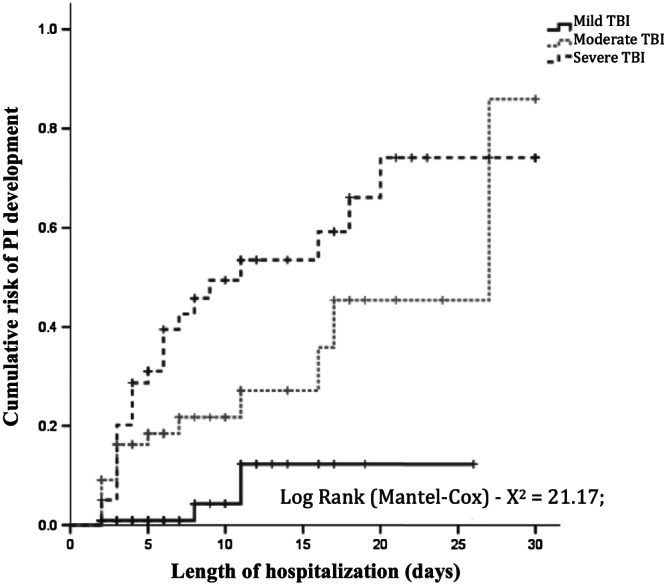
Kaplan‐Meyer curve showing a cumulative risk of PI development in patients diagnosed with TBI classified by severity

A total of 25 (55%) patients in the PI group developed PI in the first 5 days of hospitalization, and after 10 days, 37 (82.2%) patients had developed PI. The Kaplan‐Meier curve shows that PI development was higher during the first 10 days of admission (Figure 3)

The 45 patients with PI developed 53 wounds. The region and final stage of the pressure lesions are presented in Table 3. Sixteen (30.2%) PIs healed during the follow‐up period.

**Table 3:** Region of PI development and final stage

Region	Stage of PI						
	Stage 1 n (%)	Stage 2 n (%)	Stage 3 n (%)	Stage 4 n (%)	Unstageable n (%)	Deep Tissue n (%)	Total n (%)
Sacral	18 (41.8)	19 (44.2)	2 (4.6)	0	4 (9.3)	0	43 (81.1)
Occipital	2 (40.0)	1 (20.0)	0	1 (20.0)	1 (20.0)	0	5 (9.4)
Trocanter	0	1 (50.0)	0	0	0	1 (50.0)	2 (3.8)
Calcaneus	0	2 (100.0)	0	0	0	0	2 (3.8)
Ear	1 (100.0)	0	0	0	0	0	1 (1.9)

### 3.3 Outcome

After 30 days of hospitalization, 190 patients (79.2%) were discharged, 16 (6.7%) were transferred, and 34 (14.2%) died. Among those who died, 16 (35.5%) were patients with PI, and 18 (9.2%) were patients without PI (Likelihood ratio test = 18.30; OR 5.92; 95% CI, 2.66‐13.17; *P* < .001).

### 3.4 Multivariate analysis

A severity classification of severe TBI increased the likelihood of developing PI by 11‐fold. A moderate TBI classification increased the likelihood by 7‐fold. The use of noradrenaline increased the likelihood of developing PI by 4%, and each full year of age led to a 3% increase in the likelihood of developing PI. The model proposed in the multivariate analysis showed excellent discriminatory power, with an accuracy of 85.6% (95% CI, 80.1‐91.2) in the prediction of cases of PI development (Table 4).

**Table 4:** Multivariate analysis in the development of PI in TBI patients

Final Model	OR (95% CI)^a^	Wald	*P*	AUC
Moderate TBI	7.03 (1.86‐26.47)	8.31	<.01	0.856
Severe TBI	10.91 (2.70‐44.08)	11.27	<.01	
Noradrenaline use	1.04 (1.01‐1.07)	4.40	<.01	
Age (in full years)	1.03 (1.00‐1.05)	5.48	.01	

*Notes.* Hosmer‐Lemeshow test, *P* = .72.

a Adjusted according to absolute rest time.

## 4 DISCUSSION

Patients with TBI diagnoses develop complications during hospitalization that can prolong the length of hospital stay and lead to increases in the care team's workload, institutional costs, and the mortality rate.

Patients with TBI diagnoses in this study were mostly young and male, similar to samples of other studies evaluating TBI (Andriessen et al., 2011; Zampolini et al., 2012). The predominance of male TBI patients of a young age may be related to their involvement with motor vehicles and violence (World Health Organization, 2006). However, in the literature, no relationship was found between sex and the development of PI, even when compared with other TBI or neurological patients (Crozeta, 2009; Dhandapani et al., 2014).

Age was an important factor in patients with a TBI diagnosis, as each additional full year of age increased the likelihood of developing a PI by 3%. As the skin ages, there is a progressive decline in its characteristics that involves pH changes, less keratogenesis, fewer melanocytes, sweat gland atrophy, and changes in the elastic fibres of the dermis (Dealey et al., 2015). Van Gilder, Macfarlane, and Meyer (2008) reported that PI has been found more frequently in elderly individuals, affecting up to 78% of patients 60 years old and older (Van Gilder et al., 2008).

The skin colour of 78.8% of the population residing in the Amazon region is non‐Caucasian (Instituto Brasileiro de Geografia e Estatística, 2011). Despite the predominance of a non‐white skin colour in patients with a TBI diagnosis, there was no difference in skin colour between the groups with and without PI. Studies in patients who developed PI have indicated a 38% (Van Gilder et al., 2008) to 78.9% (Hyun et al., 2013) predominance of white skin colour. In white skin structures, the stratum corneum has fewer layers, which reduces resistance to external trauma (Alchorne & Abreu, 2008) and renders the skin more susceptible to PI development.

Some studies indicate that neurological patients have PI incidence rates ranging from 3.8% (Klein, Mulkey, Bena, & Albert, 2015) to 12.4% (Fife et al., 2001), neurosurgical patients show a 13.6% incidence of PI (Diccini, Camaduro, & Iida, 2009), and patients with a TBI diagnosis in the rehabilitation phase show a PI prevalence of 26.1% (Zampolini et al., 2012). In our study, during hospitalization, patients experienced an 18.8% incidence of PI, but when patients were analysed during an ICU stay, the incidence increased to 28.8%. Studies on the incidence of PI among hospitalized patients with a TBI diagnosis are rare in the literature. Dhandapani et al. (2014) evaluated patients classified with severe TBI and with GCS scores ranging from 4 to 8 points, aged 20 to 60 years old, during 21 days of hospitalization; the incidence of PI was 16%. With different inclusion criteria, the incidence of PI in patients with a diagnosis of severe TBI in our study was higher (42.6%). Comparing studies may be difficult because of the differences in criteria. In our research, we included patients with a GCS of 3 to 8 points, had no age limit, and performed the evaluation in patients who had been hospitalized for up to 30 days, which may be related to this difference in incidence.

The incidence of PI progressively increased according to TBI severity classification, with moderate or severe TBI being strong predictors of PI in our study. Patients classified as having moderate or severe TBI are at greater risk of developing intracranial hypertension. An increase in ICP can lead to changes in cerebral blood flow and neuronal destruction and, consequently, to an increase in secondary injuries (Alali et al., 2013; Algattas & Huang, 2014). This complication can cause decreased consciousness and motor changes, with a loss of mobility and sensitivity. These changes make it difficult for the patient to feel or react to discomfort caused by pressure on bony prominences, leading to an increased risk of PI (Fernandes & Caliri, 2008).

Hypotension can cause a reduction in cerebral blood flow in patients with impaired autoregulation, thereby affecting the outcome and mortality of patients diagnosed with TBI (Haddad & Arabi, 2012; Subhas & Appleby, 2011; Thompson, 2012). To maintain adequate cerebral blood flow, the use of vasopressors is recommended to maintain an SBP above 90 mmHg (Alali et al., 2013; Algattas & Huang, 2014; Subhas & Appleby, 2011). Noradrenaline is the vasopressor drug most commonly prescribed in TBI. This drug has peripheral vasoconstriction action that can decrease the supply of oxygen and nutrients to the skin and is related to the development of PI (Cox, 2013). In our study, 32 (45.1%) patients who developed PI used noradrenaline, and this use was strongly associated with PI development. Cox (2011) reported that 49% of ICU patients who developed PI used noradrenaline and had longer drug infusion times than patients without PI (*P* < .1).

The Braden Scale has proven to be a strong predictor of PI in the literature (Cox, 2011; Fernandes & Caliri, 2008; Fife et al., 2001; Hyun et al., 2013). A relationship between low Braden Scale values and the development of PI has been established, especially in patients classified as having severe TBI. However, in multivariate analysis, Braden Scale values were not associated with PI risk factors in TBI. The number of patients with mild TBI likely affected this result, as low GCS values were associated with low Braden Scale values and with a greater risk of developing PI (Fernandes & Caliri, 2008).

Several factors are involved in skin changes that lead to PI development. In this research, the factors found to be strong predictors of PI development were a TBI classification of moderate or severe, noradrenaline use, and older age. The risk factors in this study were different from those found in Dhandapani et al. (2014) study that included only severe TBI patients, which stated decreased haemoglobin values and enteral feeding commencing after more than 7 days as risk factors (Dhandapani et al., 2014). Fife et al. (2001) reported that in neurological patients, the risk factors were a Braden Scale score ≤ 13 points and low body weight according to BMI (Fife et al., 2001). This divergence in results indicates that there is a need for further studies with more homogeneous samples.

The care given to patients regarding PI development prevention should be the same as for all patients diagnosed with TBI as that offered to critical patients, since the pathophysiological changes that may occur, especially in severe and moderate TBI, can lead to an intensive care scenario. This care should include an appropriate support surface for patients, to help with changes in perfusion and oxygenation, along with movement restrictions in bed. Repositioning in bed should be individualised according to patient tolerance and stability. Small changes in position can be tolerated by patients and can allow perfusion at pressure points to be maintained without changing clinical conditions. It is also recommended that the possibility of resuming routine repositioning, along with heel pressure relief with the use of full leg supports, should be evaluated every 8 hours (National Pressure Ulcer Advisory Panel, European Pressure Ulcer Advisory Panel, & Pan Pacific Pressure Injury Alliance, 2014).

This study is important for the identification of the incidence of PI and the risk factors involved in patients with TBI diagnosis. Special attention should be directed towards patients classified as having severe TBI, whose incidence of PI is twice that of those with moderate TBI. The use of noradrenaline and older age increased the risk of PI development in patients with TBI and required the implementation of specific protocols directed towards this complication.

### 4.1 Limitations

The limitations of this study include the difficulty of applying the PI prevention protocol in the emergency room, where patients were kept on a rigid board and in a cervical collar, which may have caused a high incidence of PI. The difficulty of patient stratification according to TBI severity classification and the evaluation of risk factors in each group was also a limitation. The change in PI classifications, with the addition of two new definitions, during the execution of the research also made updating patient stratification difficult.

## 5 CONCLUSION

There was a high incidence of PI in patients diagnosed with TBI, especially in those classified as having moderate to severe TBI. Risk factors for the development of PI were a severity classification of moderate to severe TBI, noradrenaline use, and older age. Study findings can be used to inform development of nursing policy and practice development to prevent PI development in this vulnerable patient population.

## CONFLICT INTEREST

The authors have no conflict of interest to declare, and there has been no significant financial support for this work that could have influenced its outcome.

## AUTHORSHIP STATEMENT

SLO and SD were responsible for conception and design of the study. SLO collected the data. SLO and SD analysed and interpreted the data, prepared the manuscript, and approved the final version for submission. The authors confirm that meet the authorship criteria and that all authors are in agreement with the content of the manuscript.
